# Effect of Heat Treatment Temperature on Martensitic Transformation and Superelasticity of the Ti_49_Ni_51_ Shape Memory Alloy

**DOI:** 10.3390/ma12162539

**Published:** 2019-08-09

**Authors:** Peiyou Li, Yongshan Wang, Fanying Meng, Le Cao, Zhirong He

**Affiliations:** School of Materials Science and Engineering, Shaanxi University of Technology, Hanzhong 723001, China

**Keywords:** Ti–Ni alloy, shape memory alloy, martensitic transformation, superelasticity

## Abstract

The martensitic transformation and superelasticity of Ti_49_Ni_51_ shape memory alloy heat-treatment at different temperatures were investigated. The experimental results show that the microstructures of as-cast and heat-treated (723 K) Ni-rich Ti_49_Ni_51_ samples prepared by rapidly-solidified technology are composed of B2 TiNi phase, and Ti_3_Ni_4_ and Ti_2_Ni phases; the microstructures of heat-treated Ti_49_Ni_51_ samples at 773 and 823 K are composed of B2 TiNi phase, and of B2 TiNi and Ti_2_Ni phases, respectively. The martensitic transformation of as-cast Ti_49_Ni_51_ alloy is three-stage, A→R→M_1_ and R→M_2_ transformation during cooling, and two-stage, M→R→A transformation during heating. The transformations of the heat-treated Ti_49_Ni_51_ samples at 723 and 823 K are the A↔R↔M/A↔M transformation during cooling/heating, respectively. For the heat-treated alloy at 773 K, the transformations are the A→R/M→R→A during cooling/heating, respectively. For the heat-treated alloy at 773 K, only a small thermal hysteresis is suitable for sensor devices. The stable *σ*_max_ values of 723 and 773 K heat-treated samples with a large *W*_d_ value exhibit high safety in application. The 773 and 823 K heat-treated samples have large stable strain–energy densities, and are a good superelastic alloy. The experimental data obtained provide a valuable reference for the industrial application of rapidly-solidified casting and heat-treated Ti_49_Ni_51_ alloy.

## 1. Introduction

The Ni-rich Ti–Ni-based alloys are favored by researchers of engineering materials, functional materials, and biomedical alloys, based on high strength, low density, superelasticity, and shape memory properties [1,2,3,4,5]. These excellent properties enable Ti–Ni-based shape memory alloys (SMAs) to be a functional material which integrates sensing and driving [6,7]. The applications of Ti–Ni-based SMAs mainly include microelectromechanical systems and novel medical devices [6,7]. In microelectromechanical systems, the functional microgrippers, fluid-flow valves, and micromirror actuators are fabricated by using the Ti–Ni-based alloy; in the medical field, the Ti–Ni-based alloy can be successfully used in arterial stents and surgical tools [6,7]. In fact, Ti–Ni-based alloys can be used in different fields because of their shape memory or superelastic properties. SMA with martensitic phase has good shape memory effect, that is, the alloy with a specific composition can be loaded and deformed in martensitic phase state, and retain its deformed shape after unloading, and then the deformed alloy can be restored to the shape before loading when heated to the parent phase state [8]. SMA with the parent phase state has good superelasticity, that is, the strain produced by a specific component alloy under the action of external force exceeds its elastic limit variable, after unloading, the alloy can spontaneously restore to its original shape [9,10,11], and its stress-strain curve presents as non-linear. In addition, the Ti–Ni-based SMA has high damping properties, biocompatibility, and corrosion resistance, and has been applied in aerospace, machinery manufacturing, transportation, civil construction, energy engineering, biomedicine, and daily life [12,13,14,15].

To apply Ti–Ni-based SMAs in different environments, it is necessary to improve the properties of Ti–Ni alloys and to consider preparation methods. Researchers reported that two methods can improve the properties of Ti–Ni SMAs; one is heat treatment [16,17,18,19,20], and the other is the addition of minor elements [13,14,15,18,19,20,21,22]. For the single-crystal Ni-50.9 at% Ti alloy, the Ti_3_Ni_4_ phases with the different sizes are precipitated in B2 matrix phase by heat treatment [17]. The reported results show that the room-temperature hardness and recoverable energy of Ti–Ni are a maximum for very small precipitate sizes (10 nm), decrease for intermediate precipitate sizes, and increase for large precipitate sizes [17]. In addition, the Ti_3_Ni_4_ precipitate (10 nm) improves the fatigue resistance of the Ti–Ni alloy, compared to the other heat treatments [16]; samples with large incoherent Ti_3_Ni_4_ precipitates (500 nm) consistently show significant dislocation activity due to mechanical cycling [16]. The reported Ti_44.5_Ni_44.5_Nb_9_Co_2_ alloy annealed at 550 °C shows the highest yield strength, a large complete recovery strain, and high recovery stress, based on the addition of Nb and Co elements [19]. For the aged Ni-rich NiTi alloys, the formation of precipitates increase their strength and alter transformation temperatures [23]. Precipitation formation is an effective method to adjust the shape memory properties of Ti–Ni-based alloys [24]. The chemical composition, volume fraction, and coherency of the formed precipitates in Ni-rich Ti–Ni alloys can influence the transformation temperatures, matrix strength, hardness, and martensite morphology [25,26,27,28]. Thus, combination of aging and precipitates makes it possible to obtain good memory behavior and hyperelastic behavior of Ni-rich Ti–Ni alloys.

In the reported preparation of Ti–Ni alloys, the shape of Ti–Ni alloys is mainly filamentous [20,29,30,31,32]. The filamentous alloys are mainly melted in a vacuum induction furnace and graphite crucible [31,32]. The ingots are made into wires by pressure forging, rotary forging, drawing, and other processes [31,32]. The obtained wires are then heat-treated to obtain materials used in different environments. To study the mechanical response of crystallographic orientation, some researchers have prepared single-crystal Ti–Ni alloys [16,17,18,28]. Single crystals were grown by Bridgman Technology in an inert gas atmosphere. Different temperatures were used to heat-treated single crystals, and the corresponding effects of different sizes of second phase precipitated on mechanical properties were studied [16,17,18,28]. Powder metallurgy technology can produce large quantities of Ti–Ni ingots with high utilization rate of raw materials; the prepared alloys are then forged and aged to meet the needs of samples in different environments [9,33]. In addition, mechanical alloying is also a commonly used method to prepare the Ti–Ni alloy [9,34]. Ball milling time has an effect on the phase transformation and mechanical properties of the alloy [9,34]. The samples were heat treated again, and different phases were precipitated [9,33,34]. The phase transformation temperature was obtained, so that excellent mechanical properties obtained could meet the needs of engineering applications [9,33,34]. Ti–Ni alloys prepared by the above methods have many processes, or cannot be produced in batches, which increases the processing cost of Ti–Ni alloys. In fact, the preparation of Ti–Ni alloys by water-cooled copper mold casting is rarely reported. The Ti–Ni alloys prepared by rapid solidification can be molded once for some parts with a simple shape, thus shortening the preparation time and cost, and aging treatment of the prepared parts can be carried out to obtain the corresponding transformation temperature and mechanical response based on the application conditions. In this paper, the Ni-rich Ti_49_Ni_51_ alloy rods were prepared by rapid solidification technology, and the as-cast rods were heat-treated at different temperatures to gain the different contents and sizes of precipitates, which can affect the transition temperature and superelasticity of the alloy. The effect of heat treatment temperature on martensitic transformation and microstructure was investigated, and the superelastic behavior under cyclic loading was also discussed. Through these studies, the experimental data obtained provide a valuable reference for the industrial application of the rapidly-solidified casting and heat-treated Ti_49_Ni_51_ alloy.

## 2. Experimental Procedure

Various combinations of pure Ti and Ni (purity of 99.9% or higher) were used to prepare ingot of alloy with nominal compositions of Ti_49_Ni_51_ (at%) under a high vacuum (3 × 10^−3^ Pa) using a magnetically-controlled tungsten arc-melting furnace (Shenyang Scientific Instruments Co., Ltd. of Chinese Academy of Sciences, Shenyang, China) in an argon atmosphere. These raw materials were obtained from Beijing Xing rong yuan Technology Co., Ltd. (Beijing, China). The raw materials Ti and Ni were in bulk, and the range of sizes was from 5 × 5 × 5 mm^3^ to 20 × 20 × 20 mm^3^. The calculated quantities of raw materials were weighed using an analytical balance with a precision of 0.1 mg. After mixing the raw materials, a total of 30 g of raw materials were put into a water-cooled crucible to smelt into alloy ingots. To ensure the uniformity of the chemical composition of the ingots, the ingot was smelted more than 4 times. The melted ingot was placed in a copper crucible for suction casting, melted again, and then sucked into a water-cooled copper abrasives under the action of pressure difference, forming a cylindrical rod with a diameter of 3 mm and a length of 50 mm. Based on expression of cooling rate $\overset{\cdot }{T}(K/s)=10/R^{2}$, (*R* is diameter, cm) [35], the cooling rate of a rod with a diameter of 3 mm is 111 K·s^−1^. The heat treatment temperatures were set to 723, 773, and 823 K, and the heating rate was 20 K min^−1^. When heated to the set temperature, the holding time was 30 min. Then the heat-treated sample was cooled to room temperature by furnace.

The samples for mechanical and structural analysis were cut from the as-cast and heat-treated samples by using a slow steel saw. The surfaces of thin sheets with diameter of 3 mm were polished using standard metallographic procedures, consisting of grinding up to 2000 grit with SiC paper and polishing with a colloidal silica suspension. For observing the microstructure, the surfaces of polished samples need to be etched. This was accomplished with a mixed solution of HF, HNO_3_, and H_2_O, and the corresponding volume ratio was 1:4.5:4.5. The microstructures of the prepared samples were observed by optical microscope (OM, Shanghai Changfang Optical Instrument Co., LTD, Shanghai, China). The phases of samples with a diameter of 3 mm were analyzed using an X-ray diffractometer (XRD, Rigaku Company, Tokyo, Japan), at an operating voltage of 30 kV, using Cu-Kα radiation. The transformation temperatures of the samples weighing 20–50 mg were measured by differential scanning calorimetry (DSC) with heating and cooling rates of 0.33 K·s^−1^, using a Perkin Elmer DSC 7 instrument (PerkinElmer, Waltham, MA, USA). The cylindrical Ti–Ni samples with a diameter of 3.0 mm and height of ~5.0 mm were prepared by subjecting them to uniaxial compression testing at room temperature. The uniaxial compression testing was performed at room temperature using a CMT5105 electronic testing machine (Metis Industrial Systems (China) Co., Ltd., Shanghai, China), at a strain rate of 2.5 × 10^−4^ s^−1^. The quantitative data were all measured for three times, and the reported data are the average values. The cycle loading and unloading experiments were also carried out on this instrument at a strain rate of 2.5 × 10^−4^ s^−1^.

## 3. Results and Discussion

### 3.1. Microstructure of Ti_49_Ni_51_ Alloy

**Figure 1:**
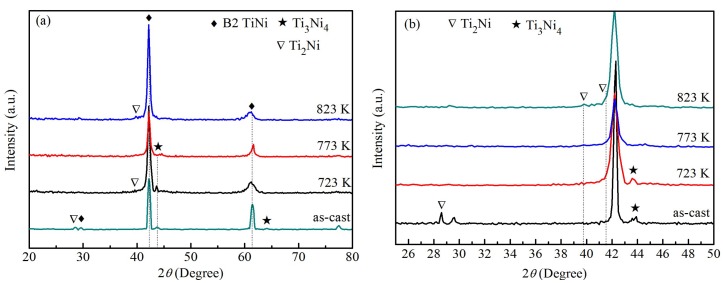
XRD patterns of the as-cast and heat-treated Ti_49_Ni_51_ samples at the different heat treatment temperatures. (**a**) 20°–80°; (**b**) 25°–50°.

Figure 1 shows the XRD patterns of as-cast and heat-treated Ti_49_Ni_51_ samples. The results show that the microstructures of as-cast and heat-treated Ti_49_Ni_51_ samples at 723 K are composed of B2 TiNi phase (CsCl structure), Ti_3_Ni_4_ phase (Rhombohedral structure), and Ti_2_Ni phase (Face-centered cubic structure). However, the microstructures of the heat-treated Ti_49_Ni_51_ samples at 773 and 823 K are composed of B2-type TiNi phase, and of B2-type TiNi phase and Ti_2_Ni phase, respectively; the diffraction peaks of Ti_3_Ni_4_ phase are not observed in Figure 1a. The weak relative diffraction intensity of the Ti_3_Ni_4_ phase indicates the low content of Ti_3_Ni_4_ phase. Accordingly, the content of the Ti_3_Ni_4_ phase of heat-treated samples is lower than that of the as-cast alloy. To clearly see the diffraction peak of Ti_2_Ni phase, Figure 1b is a local enlargement of Figure 1a. The Ti_2_Ni diffraction peak of as-cast alloys is mainly below 30°. For the 723 K heat-treated alloy, the diffraction peak of Ti_2_Ni phase is 39.6°; for the 773 K heat-treated alloy, Ti_2_Ni and Ti_3_Ni_4_ phases are not found in XRD patterns. When the heat treatment temperature is further increased, a small amount of Ti_2_Ni precipitates from the 823 K heat-treated alloy. Because the diffraction angle of the strongest diffraction of Ti_2_Ni phase is 41.68°, the diffraction peaks of the TiNi phase in 723 and 823 K heat-treated alloys become wider, as shown in Figure 1b. Because the intensity of the main diffraction peak of Ti_2_Ni phase is weak, the results show that the content of the Ti_2_Ni phase is relatively small. The cause of Ti_2_Ni phase precipitation needs to be further studied in future work.

**Figure 2:**
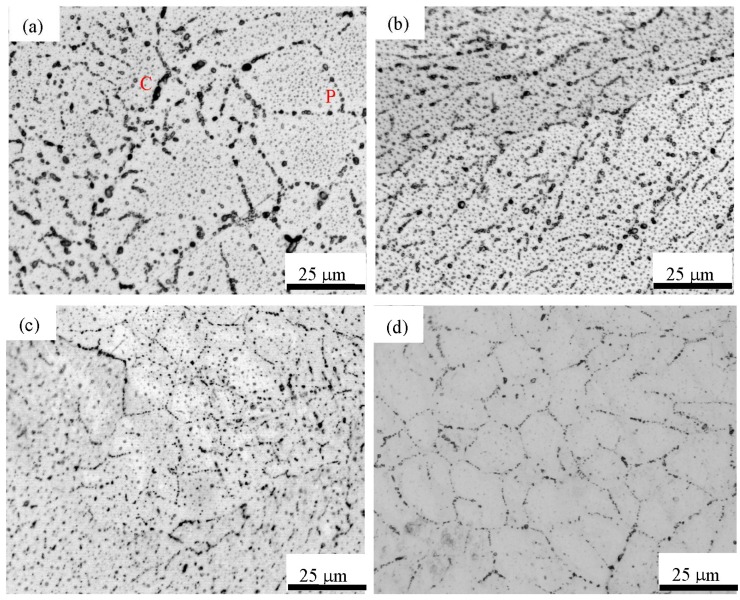
Microstructure of as-cast and heat-treated Ti_49_Ni_51_ samples at the different temperatures. (**a**) As-cast, (**b**) 723 K, (**c**) 773 K, and (**d**) 823 K.

Figure 2 shows the microstructure of as-cast and heat-treated Ti_49_Ni_51_ samples. The results show that a large number of Ti_3_Ni_4_ phases precipitate along the grain boundary of B2 TiNi phases, and a small amount of Ti_3_Ni_4_ phases precipitate inside B2 TiNi phases. Because of the small content of Ti_2_Ni phase, it is difficult to distinguish Ti_2_Ni from Ti_3_Ni_4_ particles in Figure 2. Therefore, in discussing Figure 2, the precipitation of Ti_2_Ni phase in TiNi phase is neglected. In addition, the content of Ti_3_Ni_4_ phase for the as-cast Ti_49_Ni_51_ alloy (see Figure 2a) is significantly higher than that for the heat-treated Ti_49_Ni_51_ alloy (see Figure 2b–d). In addition, the content and size of Ti_3_Ni_4_ phase decrease with the increase of heat treatment temperature, as shown in Figure 2b–d. The grain sizes of Ti_3_Ni_4_ phase for the as-cast Ti_49_Ni_51_ alloy (see Figure 2a) are also larger than those of the heat-treated Ti_49_Ni_51_ alloy (see Figure 2b–d). In fact, the range of size of Ti_3_Ni_4_ particles in the as-cast alloy is 0.2–7 µm; for the 723 K heat-treated alloys, the range of size of Ti_3_Ni_4_ particles is 0.2–3 µm; for the 773 and 823 K heat-treated alloys, the ranges of size of precipitates are 0.2–2 µm and 0.2–1 µm, respectively.

Accordingly, the range of size of precipitates decrease with the increase of heat treatment temperatures. The microstructures in Figure 2 show that the grains of TiNi phase are surrounded by the continuous or discontinuous layers of Ti_3_Ni_4_ phase. This phenomenon is connected with the so-called complete and incomplete wetting of grain boundaries by the second solid phase both in the Ti–Fe(Co) and Ti–Fe–Sn alloys [36,37,38]. A large and/or long grain of Ti_3_Ni_4_ phase between two β-Ti phases form continuous layers, and this phenomenon is called complete grain boundary wetting (labeled in Figure 2a by the letter C); while multiple adjacent Ti_3_Ni_4_ phases precipitated on grain boundaries form the discontinuous layers, corresponding to the incomplete or partial wetting of grain boundary (labeled in Figure 2a by the letter P). Therefore, in Figure 2, the Ti_3_Ni_4_ phase forms the complete and incomplete grain boundary wetting.

### 3.2. Martensitic Transformation Temperature of Ti_49_Ni_51_ Alloy

In the practical aspect of Ti–Ni SMAs, a low transformation temperature can make pipe joints, and a small thermal hysteresis can make elastic and vibrator [8]. According to the different heat-treatment conditions, Ti–Ni SMAs can undergo one-stage, two-stage, or three-stage transformation. One-stage transformation occurs mostly from the parent phase (A), with simple cubic structure (CsCl), to martensitic phase (M), with monoclinic structure (A→M); and also from A to R phase with rhomboidal structure (A→R) [39]. The two-stage transformation is from A to R phase, and then to M phase, that is, A→R→M transformation. The three-stage transformation can be divided into two cases: One is one-stage R-phase plus two-stage M transformation (R→M1→M2); the other is two-stage R phase plus one-stage M transformation (R1→R2→M) [39]. 

**Figure 3:**
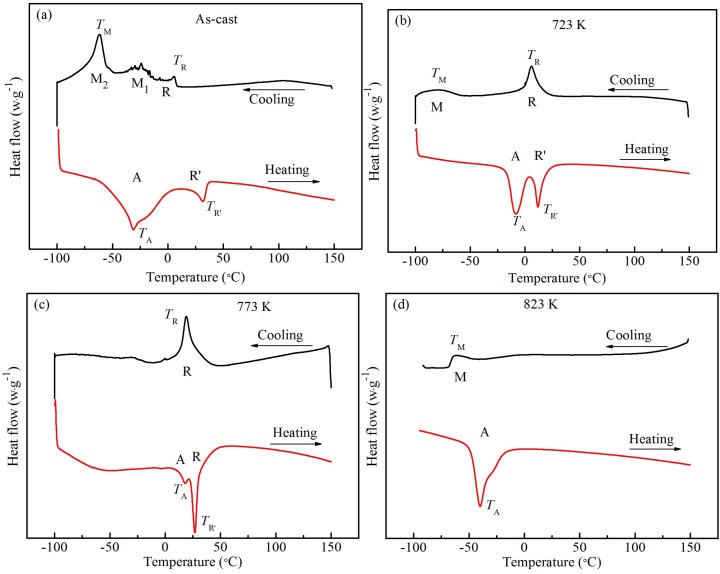
DSC curves of as-cast and heat-treated Ti_49_Ni_51_ samples. (**a**) As-cast, (**b**) 723 K, (**c**) 773 K, (**d**) 823 K.

Figure 3 shows the DSC curves of as-cast and heat-treated Ti_49_Ni_51_ samples. In Figure 3, M and A represent martensite transformation and reverse martensite transformation, respectively; R and R′ represent R-phase and reverse R-phase transformation (rhomboidal structure), respectively. The peak temperatures of transformation during cooling and heating, *T*_M_ and *T*_A_, represent the martensite and reverse martensite transformation temperatures, respectively. The peak temperatures of R-phase transformation during cooling and heating, *T*_R_ and *T*_R′_, represent R-phase and inverse R-phase transformation temperatures, respectively. The measured *T*_M_, *T*_A_, *T*_R_, and *T*_R′_ values are list in Table 1. In Figure 3a, for the as-cast Ti_49_Ni_51_ alloy, the transformation types are three-stage A→R, R→M_1_ and R→M_2_ transformation during cooling, and the two-stage A→R→M martensitic transformation occurs during heating. In fact, during the heating, the inverse transformation of the subsequent-formed martensite phase in cooling first occurs, that is, the inverse transformation of M_2_ phase occurs first, compared with the M_1_ phase [39]. Due to the inconsistency of the inverse transformation temperature between M_2_→R and M_1_→R, the inverse transformation peak becomes wide, or the temperature range becomes wide, as shown in Figure 3a. In Figure 3b, for the heat-treated Ti_49_Ni_51_ alloy at 723 K, the two-stage A→R→M martensitic transformation occurs in the cooling stage; however, the two-stage reverse martensitic transformation is M→R→A in the heating stage. In the cooling, the *T*_R_ values of the R-phase transformation temperatures are 278.8 K for the as-cast alloy, and 279.1 K for the heat-treated alloy at 723 K, and approximately the same; however, in the heating stage, the *T*_R’_ values of the R-phase inverse transformation temperature is 304.9 and 285.1 K, respectively, and the *T*_R’_ value of the heat-treated alloy is shifted to the low temperature, comparing with that of the as-cast alloy. The *T*_M_ values of as-cast alloy and heat-treated alloy at 723 K are 211.4 and 189.1 K, respectively, which indicates that the *T*_M_ of the heat-treated alloy moves to a low temperature during the cooling. But, in the heating, the reverse martensitic transformation temperature increases from 241.8 K for the as-cast alloy to 265.3 K for the heat-treated alloy at 723 K. In Figure 3c, for the heat-treated Ti_49_Ni_51_ alloy at 773 K, the transformation type of the alloy during the cooling stage is the one-stage A→R transformation, while during heating, the inverse martensitic transformation is similar to the two-stage transformation of M→R→A. In Figure 3d, for the heat-treated Ti_49_Ni_51_ alloy at 823 K, the phase transformation types of the alloy during cooling and heating are the one-stage A→M and M→A transformation, respectively. For the heat-treated Ti_49_Ni_51_ alloy at 773 K, during the cooling, the *T*_R_ value of A→R is 292.2 K, which is higher than those of the as-cast alloy and heat-treated alloy at 723 K (Seen Table 1 and Figure 2). The *T*_R′_ value is equal to 300 K, which is larger than that of the heat-treated alloy at 723 K, but smaller than that of the as-cast alloy.

The A→R transformation is the transition of the parent phase to a martensite phase, and it is a transition process of a simple cubic structure (BCC) with a higher symmetry to a lower symmetrical rhombohedral structure; however, the R→M phase transformation is converted to a martensitic to martensitic transformation process (i.e., the symmetry of the lower rhombohedral structure to the transition of the monoclinic structure with a lowest symmetry) [31]. For the as-cast and the heat-treated samples at 723 and 773 K, the A→R transformation fist occurs during the cooling. The reason is that the symmetry of the R-phase with the rhombohedral structure is higher than that of the monoclinic structure of M phase. When the temperature is further reduced, the R-phase transformation is shifted towards a monoclinic-structural martensitic phase with a lower symmetry, comparing with the symmetry of R phase (i.e., the R→M transformation). For the heat-treated alloy at 823 K, the content of Ti_3_Ni_4_ phase precipitate inside and at the grain boundary of B2 phase is obviously lower than those of the as-cast alloy and the heat-treated alloy at 723 and 773 K, as shown in Figure 2. Accordingly, the microstructure of the heat-treated alloy at 823 K is in the state of recrystallizing, which results in the decrease of dislocation density, the decrease of martensitic transformation resistance [31,39], and the increase of transformation peak position. It indicates that the *T*_M_ value of the heat-treated alloy at 823 K is higher than that of the heat-treated alloy at 723 K. When the heat treatment temperature is high, the density of residual defect in the alloy decreases, the microstructure uniformity improves, and the effective position of R-phase nucleation decreases, which leads to the delay of R-phase transformation or the decrease content of R-phase transformation [31,39]. The R-phase transformation is not easy to be detected on the DSC curve. Accordingly, the one-stage transformation occurs in heat-treated Ti_49_Ni_51_ alloy at 823 K (i.e., the one-stage transformation of A→M and M→A).

The as-cast alloy and heat-treated alloy at 723 K exhibit high residual dislocation density, large residual stress, and more residual texture [31,39]. The interaction of these structural defects with the stress field of M transformation can inhibit the martensitic phase transformation, which results in that the martensitic transformation is delayed (i.e., the *T*_M_ value becomes low) [20,26]. In addition, as the stress field generated from the R-phase transformation is weaker than that generated from the M-phase transformation, the effect of the structural defect on the stress field of the R-phase transformation is small, and the inhibition of the A to R phase transformation is reduced [31,39]. Accordingly, the R-phase transformation takes place preferentially, that is, the *T*_R_ value is larger than the *T*_M_ value. These will result in the separation of the R-phase and the M-phase, as shown in Figure 3a,b.

For superelastic alloys, thermal hysteresis (Δ*T*) of phase transformation is a thermodynamic performance index in industrial applications [39]. The Δ*T* value is the temperature difference of the positive and negative peaks of transformation, which indicates the width of range of the operating temperature for the devices made of superelastic alloys [39]. The larger the Δ*T* value is, the wider the operating temperature range of the device is. The alloy with the larger Δ*T* value is suitable for making the connection elements, and the alloy with the smaller Δ*T* value is suitable for making sensors [39]. For the heat-treated alloy at 723 K, the thermal hysteresis (Δ*T*_M_) of M-phase transformation (76.2 K) is larger than that of the as-cast alloy (30.4 K), due to the decrease of the martensitic transformation temperature during the cooling, and the increase of the reverse transformation temperature during the heating for the heat-treated alloy at 723 K. For the heat-treated Ti_49_Ni_51_ samples at 723 and 773 K, the thermal hysteresis (Δ*T*_R_) values of the R-phase transformation are 6.0 and 7.8 K, respectively, which are larger than that of as-cast alloy. The difference of two Δ*T*_R_ values for the heat-treated samples at 723 and 773 K is small, which indicates that the thermal hysteresis of R-phase transformation has good stability at the different heat treatment temperatures. In fact, the as-cast alloy and heat-treated alloy at 723 K have the larger thermal hysteresis, which are suitable for bonding devices. For the heat-treated alloy at 773 K, only a small thermal hysteresis is suitable for sensor devices. For the 823 K heat-treated Ti_49_Ni_51_ alloy, only a large thermal hysteresis is not only suitable for making junction devices, but also suitable for making sensor devices with a wide temperature range.

### 3.3. Mechanical Properties of the Ti_49_Ni_51_ Alloy

**Figure 4:**
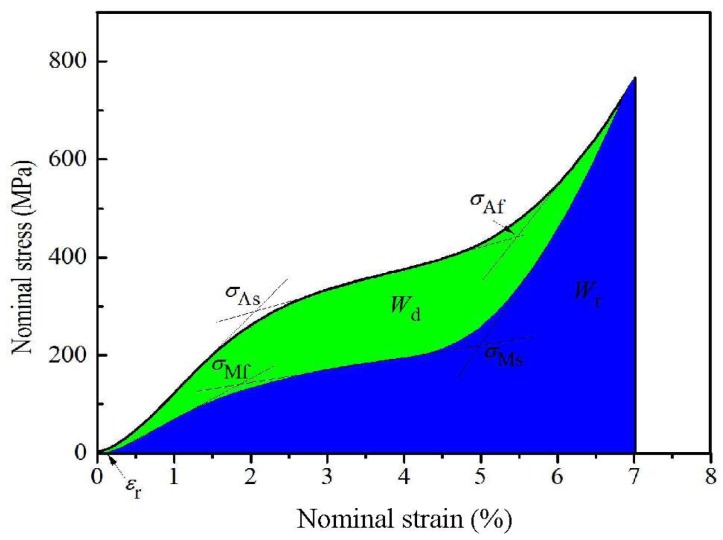
The first cyclic loading/unloading nominal stress-strain curve of the heat-treated Ti_49_Ni_51_ alloy (723 K) at the strain of 7%.

Figure 4 shows the first cyclic loading/unloading stress-strain curve of the heat-treated Ti_49_Ni_51_ alloy (723 K) at a strain of 7%. In Figure 4, the initial (*σ*_As_) and finishing (*σ*_Af_) stress of martensitic transformation, the initial (*σ*_Ms_) and the finishing (*σ*_Mf_) values of reverse martensitic transformation are labeled by the method of two tangents. The area defined by the unloading stress-strain curve and the strain is defined as the recoverable strain energy density (*W*_r_); the area enclosed by the load stress-strain curve and the unloading stress-strain curve is defined as the dissipation energy (*W*_d_); in the unloading process, the strain is defined as the residual strain (*ε*_r_) when the stress is equal to zero, as shown in Figure 4.

**Figure 5:**
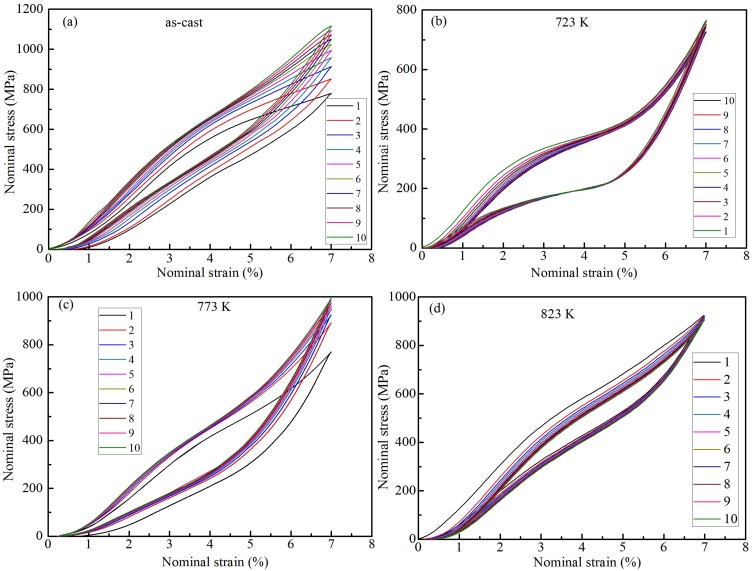
Cyclic loading/unloading nominal stress-strain curves of as-cast and heat-treated Ti_49_Ni_51_ samples at a strain of 7%; (**a**) As-cast, (**b**) 723 K, (**c**) 773 K, and (**d**) 823 K.

Figure 5 shows the cyclic loading/unloading nominal stress-strain curves of as-cast and heat-treated Ti_49_Ni_51_ samples at a strain of 7%. In the loading process of the as-cast Ti_49_Ni_51_ alloy, the *σ*_As_ values of martensitic transformation are exhibited in Figure 5a, but the *σ*_Af_ values of martensitic transformation are not found in Figure 5a; during unloading, the stress platform of reverse martensitic transformation is not presented. For the heat-treated samples at 723 and 823 K, the stress transformation platform are exhibited in the loading and unloading processes, and the *σ*_As_ and *σ*_Af_ values can be presented in Figure 5b,d; the *σ*_Ms_ and *σ*_Mf_ values of reverse martensitic transformation can be presented during unloading. For the heat-treated alloy at 773 K, the stress transformation platform is obvious in the loading process, which indicates that the *σ*_As_ and *σ*_Af_ values can be distinguished; while in the unloading process, the strain transformation platform is not observed in Figure 5c, but the *σ*_Ms_ value can be calculated. 

**Figure 6:**
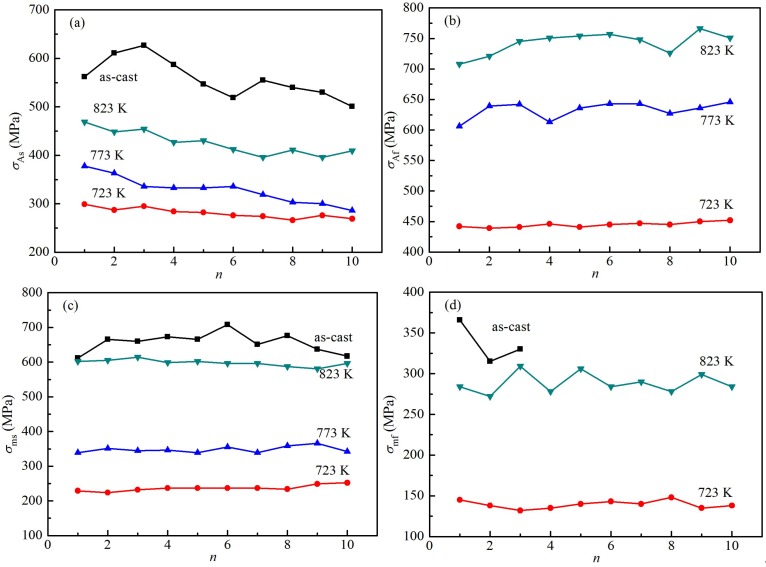
The relation of *σ*_As_, *σ*_Af_, *σ*_ms_, *σ*_mf_ and cyclic loading/unloading number (*n*). (**a**) *σ*_As_ and *n*; (**b**) *σ*_Af_ and *n*; (**c**) *σ*_ms_ and *n*; (**d**) *σ*_mf_ and *n*.

The relation of *σ*_As_, *σ*_Af_, *σ*_ms_, *σ*_mf_ values calculated from Figure 4 and cyclic loading/unloading number *(n*) is shown in Figure 6. In Figure 6a, the *σ*_As_ values for the as-cast Ti_49_Ni_51_ alloy vary greatly with the *n* values, while the *σ*_As_ values of the heat-treated alloy change slightly. In addition, at the same loading times, the *σ*_As_ values of the as-cast Ti_49_Ni_51_ sample are larger than those of the heat-treated Ti_49_Ni_51_ samples at three temperatures, but the *σ*_As_ values of the heat-treated Ti_49_Ni_51_ samples increase with the increase of the heat treatment temperatures, as shown in Figure 6a. In Figure 6b, the *σ*_Af_ values of the heat-treated alloy at 723 K are approximately unchanged. Most of the *σ*_Af_ values of the heat-treated alloy at 773 K are similar, except for a small amount of *σ*_Af_ values. The *σ*_Af_ values of the heat-treated alloy at 823 K change slightly from the third to the seventh time, while the *σ*_Af_ values of the others change greatly. In fact, the *σ*_Af_ values increase with the increase of the heat treatment temperatures. 

Due to the high density of residual dislocation, large residual stress, and large residual texture of the as-cast alloy, the resistance of M-phase transformation of the B2 TiNi structure increases under compressive stress [31], which leads to the larger initial stress and finishing stress of martensite transformation, comparing to the heat-treated alloy. Although R-phase transformation exists in as-cast alloys, it can be seen from Figure 3 that the enthalpy of R-phase transformation is obviously smaller than that of M-phase transformation. Accordingly, the M-phase transformation is the main phase transformation in the compression process, and a small amount of R-phase transformation does not significantly reduce the initial and finishing stress of martensitic transformation. For the 723 and 773 K heat-treated samples, the dislocation density, residual stress and residual texture decrease [31], and the main R-phase transformation occurs during the cooling process. When the compressive stresses are applied to the heat-treated samples at 723 and 773 K, the main R-phase transformation occurs. However, the stress field generated from the R-phase transformation is weaker than that generated from the M-phase transformation [31], which results in that the *σ*_As_ and *σ*_Af_ values of the heat-treated samples at 723 and 773 K are lower than the corresponding values of the as-cast sample. For the heat-treated alloy at 823 K, as the alloy is in recrystallization state, the effective position of R-phase nucleation decreases [31], and only M-phase transformation occurs, as shown in Figure 3d. As the stress field generated from the M-phase transformation is larger than that generated from the R-phase transformation [31], the M-phase transformation stress is higher than the R-phase transformation stress under compressive stress, which explains that the *σ*_As_ and *σ*_Af_ values of the heat-treated sample at 823 K are higher than the corresponding values of heat-treated samples at 723 and 773 K.

In Figure 6c, the *σ*_Ms_ values of the as-cast alloy vary greatly, while the *σ*_Ms_ values of the heat-treated alloy are approximately unchanged, while the *σ*_Ms_ values of the as-cast alloy are higher than that of *σ*_Ms_ values of the heat-treated alloy. In addition, the *σ*_Ms_ values of the heat-treated alloy increase with the increase of heat treatment temperatures. In Figure 6d, the as-cast alloy only shows the three *σ*_Mf_ values from first unload to third unload, but the *σ*_Mf_ values of the heat-treated alloy at 773 K are not shown in Figure 5c. The variation of *σ*_Mf_ values are small for the heat-treated alloy at 723 K, but the variation of *σ*_Mf_ values fluctuate for the 823 K heat treatment alloy. The *σ*_Mf_ values of the heat treatment alloy at 823 K are higher than those of the heat-treated alloy at 723 K, as shown in Figure 6d. In fact, the as-cast and 823 K heat-treated samples have relatively large changes in stress of martensitic transformation, and weak relative stability, which indicates that the as-cast and 823 K heat-treated samples are the hyperelastic materials with poor stability; the stress of the martensitic transformation for the heat-treated samples at 723 and 773 K changes slightly, and have relatively high stability, which indicates that the two heat-treated samples are the hyperelastic materials with good stability.

**Figure 7:**
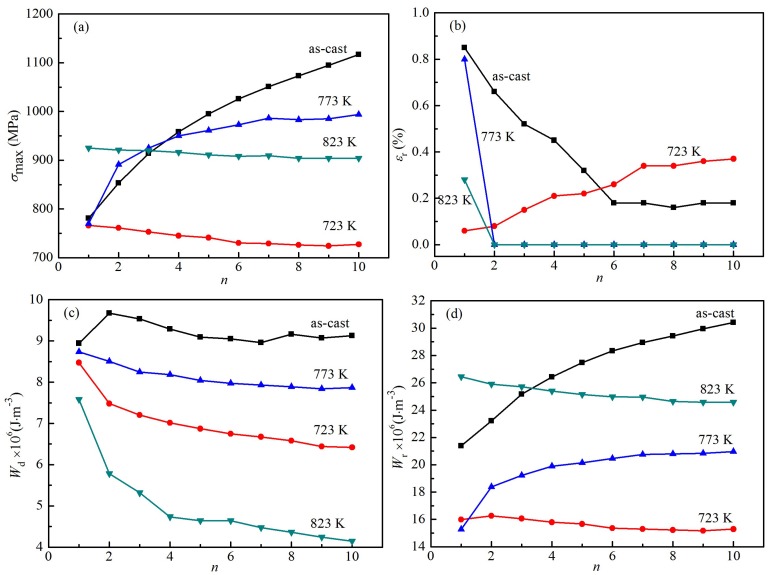
The relation between the *σ*_max_, *ε*_r_, *W*_d_, *W*_r_, and cyclic loading/unloading number (*n*). (**a**) *σ*_max_ and *n*; (**b**) *ε*_r_ and *n*; (**c**) *W*_d_ and *n*; (**d**) *W*_r_ and *n*.

Figure 7 shows the relation between the calculated *σ*_max_, *ε*_r_, *W*_d_, *W*_r_ values, and cyclic loading/unloading number (*n*). In Figure 7a, the *σ*_max_ values of the as-cast alloy increase with the increase of loading times, increasing from 781 MPa in the first cycle to 1117 MPa in the tenth cycle, and increasing approximately linearly from the sixth cycle, resulting in that the linear increase of maximum stress can easily cause plastic deformation of the sample. For the heat-treated alloy at 773 K, with the increase of cycle times, the *σ*_max_ values increase rapidly at first, then slowly, and finally tend to be stable, which improves the safety during cyclic loading and unloading. Under the 723 and 823 K heat-treatment conditions, the *σ*_max_ values of the alloy decrease slowly, and during the whole cycle, those values decrease slightly, and finally tend to be stable. Figure 7b shows the relation between the *ε*_r_ and *n* values. For the as-cast alloy, the *ε*_r_ values decreases rapidly from the first time to the sixth time, then tends to be stable, and are less than 0.2%. The *ε*_r_ values of the heat-treated alloy at 723 K increase from 0.06% to 0.37% with the increase of cycle times. In fact, the increase is small. In addition, for the heat-treated samples at 773 and 823 K, a small *ε*_r_ value appears for the first time, and the *ε*_r_ values are equal to zero for the rest of the cycles. It shows that the content of martensitic transformation is approximately equal to that of reverse phase transformation under loading and unloading conditions. Figure 7c shows the relation between the *W*_d_ and *n* values. The *W*_d_ values of the as-cast sample are larger than those of the heat-treated samples, while the *W*_d_ values of the 823 K heat-treated sample are significantly smaller than those of the as-cast sample and heat-treated samples at 723 and 774 K. The *W*_d_ values of the 823 K heat-treated alloy begin to decrease rapidly with the increase of cycle number, and slowly decline from the fourth time; the *W*_d_ value of the 723 K heat-treated alloy decrease rapidly between the first and second time, slowly decline from the second time, and finally change slightly, and tend to be stable. The *W*_d_ values of the 773 K heat-treated alloy decrease slowly, and finally stabilize during the whole cycle. These results show that the *W*_d_ values of the heat-treated samples at 723 and 773 K are relatively stable, though smaller than those of the as-cast sample. As the *σ*_max_ values of the as-cast alloy increase gradually, the small plastic deformation occurs during the final cyclic loading process, as shown in Figure 5a. Although there are the large *W*_d_ values of the as-cast alloy, it has low safety in application. As the *σ*_max_ values tend to be stable for the 723 and 773 K heat-treated samples with a large *W*_d_ value, the safety of the heat-treated samples is higher than that of the as-cast sample in application. Because the residual strain tends to zero in the 823 K heat-treated alloy, the smaller dissipated energy can be used in the working environment, where the dissipated energy requirement is not high. 

Figure 7d shows the relation between the *W*_r_ values and the number of cycles. The *W*_r_ values of the as-cast sample increase with the increase of cycle times, and are larger than those of the heat-treated samples at 723 and 773 K. For the 823 K heat-treated alloy, the *W*_r_ values decrease slowly with the increase of cycle number, and tend to remain unchanged at last. For the 723 K heat-treated alloy, the *W*_r_ values change slightly or tend to be stable during the whole cycle. For the 773 K heat-treated alloy, the *W*_r_ values change slightly from the second time, and finally tend to be stable. Therefore, the *W*_r_ values of the heat-treated samples are more stable than those of the as-cast sample. In fact, the samples treated at 773 and 823 K have relatively stable strain–energy densities, and are a good superelastic alloy.

## 4. Conclusions

The martensitic transformation and mechanical properties of the as-cast and heat-treated Ti_49_Ni_51_ samples prepared by rapidly-solidified technology were investigated. The main results are summarized as follows:

(1) The microstructures of the as-cast and 723 K heat-treated Ti_49_Ni_51_ samples are composed of B2 TiNi phase, Ti_3_Ni_4_, and Ti_2_Ni phases; the microstructures of the heat-treated Ti_49_Ni_51_ samples at 773 and 823 K are composed of B2 TiNi phase, and of B2 TiNi phase and Ti_2_Ni phase, respectively. The content and size of Ti_3_Ni_4_ phase decrease with the increase of heat treatment temperatures.
(2) The transformation of the as-cast Ti_49_Ni_51_ sample are third-stage A→R→M_1_ and R→M_2_ during cooling, and two-stage A→R→M transformation during heating. The transformations of the heat-treated Ti_49_Ni_51_ samples at 723 and 823 K are the two-stage A↔R↔M and one stage A↔M transformation during cooling/heating, respectively. For the heat-treated sample at 773 K, the transformations are the A→R/M→R→A during cooling/heating.
(3) For the heat-treated alloy at 773 K, only a small thermal hysteresis is suitable for sensor devices. For the 823 K heat-treated alloy, only a large thermal hysteresis is not only suitable for making junction devices, but also suitable for making sensor devices with a wide temperature range.
(4) The stable *σ*_max_ values of the 723 and 773 K heat-treated samples with a large *W*_d_ value exhibit that the safety of the heat-treated samples is higher than that of the as-cast sample in application. The samples treated at 773 and 823 K have relatively stable strain–energy densities, and are a good superelastic alloy.

## Figures and Tables

**Table 1:** Thermodynamic parameters of the as-cast and heat-treated Ti_49_Ni_51_ samples in the DSC curves, including martensitic transformation temperature (*T*_M_), reverse martensitic transformation temperature (*T*_A_), R-phase transformation temperature (*T*_R_), reverse R-phase transformation temperature (*T*_R′_), thermal hysteresis of martensitic transformation (Δ*T*_M_), thermal hysteresis of R-phase transformation (Δ*T*_R_).

Alloy	*T*_M_ (K)	*T*_A_ (K)	*T*_R_ (K)	*T*_R′_ (K)	Δ*T*_M_ (K)	Δ*T*_R_ (K)
Ti_49_Ni_51_ (as-cast)	211.4	241.8	278.8	304.9	30.4	26.1
Ti_49_Ni_51_ (723 K)	189.1	265.3	279.1	285.1	76.2	6.0
Ti_49_Ni_51_ (773 K)	--	--	292.2	300	--	7.8
Ti_49_Ni_51_ (823 K)	208.4	233.2	--	--	24.8	--
